# Comprehensive assessment of SARS-CoV-2 antibodies against various antigenic epitopes after naive COVID-19 infection and vaccination (BNT162b2 or ChAdOx1 nCoV-19)

**DOI:** 10.3389/fimmu.2022.1038712

**Published:** 2022-12-12

**Authors:** Jihyun Lee, Dong-Gun Lee, Jin Jung, Ji Hyeong Ryu, Soyoung Shin, Sung-Yeon Cho, Raeseok Lee, Eun-Jee Oh

**Affiliations:** ^1^Department of Biomedicine and Health Sciences, Graduate School, The Catholic University of Korea, Seoul, Republic of Korea; ^2^Division of Infectious Diseases, Department of Internal Medicine, Seoul St. Mary’s Hospital, College of Medicine, The Catholic University of Korea, Seoul, Republic of Korea; ^3^Vaccine Bio Research Institute, College of Medicine, The Catholic University of Korea, Seoul, Republic of Korea; ^4^Department of Laboratory Medicine, Seoul St. Mary’s Hospital, College of Medicine, The Catholic University of Korea, Seoul, Republic of Korea; ^5^Resesarch and Development Institute for In Vitro Diagnostic Medical Devices of Catholic University of Korea, Seoul, Republic of Korea; ^6^Department of Laboratory Medicine, Daejeon St. Mary’s Hospital, College of Medicine, The Catholic University of Korea, Seoul, Republic of Korea

**Keywords:** COVID-19, SARS-CoV-2, vaccination, serology, multiplex-bead assay, new variants

## Abstract

Comprehensive assessment of SARS-CoV-2 antibodies against antigenic epitopes and cross-neutralization on variants is essential to monitor after infection or vaccination. From 32 COVID-19 patients and 40 vaccinated individuals [20 Oxford–AstraZeneca (AZ) and 20 Pfizer–BioNTech (BNT)], 348 serial sera are collected until 40 days after infection and 3 months after homologous booster vaccination. Antibody levels were monitored using a multiplex-bead assay including variant spike antigens, Roche (S1/RBD total) and a surrogate virus neutralization test (GenScript). Anti-S/S1/RBD levels were higher than anti-S2/N levels from 2 weeks after infection and were higher in severe infection (*P* < 0.05). Vaccination showed highest antibody levels after 1-month booster and had consistently high levels in the order of anti-full S, anti-RBD, anti-S1 and anti-S2. Infection induced higher anti-S2/N levels than prime vaccination (*P* < 0.05). Three months after BNT/BNT vaccination, antibody levels against S1/RBD and 23 variant antigens were higher than post-infection or AZ groups (*P* < 0.05). Regarding intraindividual changes from post-prime to post-boost vaccination, boost induced a 1.1- to 3.9-fold increase on multiplex-bead assay, 22.8- to 24.2-fold on Roche assay and 22.8- to 24.2-fold on GenScript assay. Post-prime levels by multiplex-bead assay predicted post-boost levels, but Roche and GenScript results were not predictive in the AZ group. The kinetics of SARS-CoV-2 antibody levels vary depending on the antigenic epitopes, assay kit, disease severity or vaccine type. Assessing seroconversion using multiplex-bead assays may contribute to monitoring the disease course, adjusting vaccination strategies, and accelerating vaccination efficacy.

## 1 Introduction

Coronavirus disease 2019 (COVID-19), which is caused by severe acute respiratory syndrome coronavirus 2 (SARS-CoV-2), has become a pandemic and presents a major health concern across the globe (1). Although vaccines have shown high levels of effectiveness against COVID-19-related diseases, the increasing prevalence of SARS-CoV-2 variants has raised concerns about reduced vaccine effectiveness (2–4). The spike protein on the surface of SARS-CoV-2 virus particles is the main target for neutralizing antibodies, and the S protein expressed through the vaccine is similar to infectious particles. Because the S protein exhibits a high degree of variability between different virus strains, a comprehensive evaluation of infection- and vaccine-derived antibodies against different antigenic epitopes and cross-neutralization on variants is important for better understanding of immune response to COVID-19 and vaccination (2, 5–8).

Several COVID-19 vaccination regimens are currently used to accelerate population coverage, and most vaccines have been developed for delivery of the spike immunogen, including mRNA, adenovirus, and protein-adjuvant platforms. In South Korea, four vaccines have been approved for use, with Pfizer BioNTech BNT162b2 (BNT) and Oxford AstraZeneca ChAdOx1 nCoV-19 (AZD1222, AZ) the most widely used (9). Both BNT and AZ vaccines deliver spike protein, but different delivery systems have potential to mediate significantly different forms of antigen presentation that may be reflected in a different humoral immune response (4, 10, 11). However, there are few real data comparing sequential antibody responses to different antigenic epitopes between natural infection, BNT and AZ vaccinations.

Antibodies against spike protein are likely to have the function of neutralizing antibodies, and numerous studies have shown a correlation between spike protein binding assays and functional virus neutralization assays (12–16). Antibodies against SARS-CoV-2 specific nucleocapsid (N) antigens are induced early and strongly in most infected individuals due to the strong immunogenicity (17). Luminex-based multiplex-bead assays are designed to identify antibody responses to multiple SARS-CoV-2 targets including full spike protein (S), individual domains of spike protein (S1, S2 and receptor binding domain (RBD)) and nucleocapsid protein. Furthermore, an additional multiplex assay was developed containing 23 additional microbeads coated with new variant spike antigens.

The aim of this study was to investigate and compare sequential SARS-CoV-2 antibody responses to different antigens using a multiplex-bead assay in COVID-19 patients and healthcare workers (HCWs) who received two doses of BNT or AZ vaccines. Antibody levels were also compared to those from a quantitative chemiluminescent immunoassay, Elecsys Anti-SARS-CoV-2 S (Roche, Basel, Switzerland) detecting S1 RBD total Ig and a surrogate virus neutralization test (sVNT, GenScript). Our data highlight the need for serological monitoring using standardized serological assays across laboratories within subpopulations for vaccine format and study participants.

## 2 Material and methods

### 2.1 Serum samples from COVID-19-infected individuals

A total of 188 serial serum samples from 32 hospitalized COVID-19 patients (16 males, 16 females, median age 63 years (range; 35-83 years)) were tested. All patients were confirmed COVID-19 positive on RT-PCR between March 2020 and December 2020 at Seoul St. Mary’s Hospital. We used serum samples from a previous study (18). Serum samples were collected during hospitalization up to 40 days after symptom onset, and were subdivided into the following groups according to days from symptom onset as described in the previous study: ≤5 days, 6-8 days, 9-11 days, 12-14 days, 15-21 days, and ≥ 22 days. The data by Roche and sVNT reported in our previous study was compared with data from a multiplex-bead assay in the current study. Disease course was classified as mild (n = 13) or severe (n = 19) as described before (18, 19). Written informed consent was waived by the board because the current study was retrospective in nature using medical records and residual serum samples (KC20SISI0879).

### 2.2 Serum samples from SARS-COV-2-vaccinated individuals

**Figure 1:**
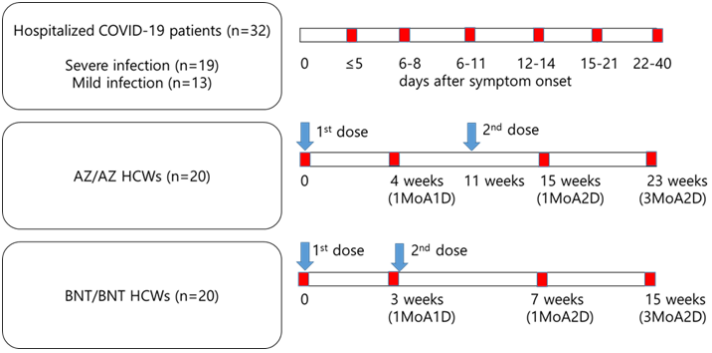
Red boxes indicate when serum samples were collected and antibodies were measured.

A second cohort included 40 HCWs with COVID-19 vaccination between March 2021 and June 2021 at Seoul St. Mary’s Hospital. Twenty subjects (2 males, 18 females, median age 40 years (range; 24-57 years)) received two doses of the AZ vaccine (AZ/AZ) 11 weeks apart. In these AZ vaccinated individuals, blood was collected before vaccination, 1 month after the first dose, and 1 month and 3 months after the 2nd dose (15 weeks and 23 weeks after the first vaccine dose). Twenty subjects (7 males, 13 females, median age 42 years (range; 24-56 years)) received two doses of the BNT vaccine (BNT/BNT) 3 weeks apart. In the BNT vaccinated individuals, blood was collected before vaccination, 1 month after the 1st dose (immediately before receiving the second vaccine dose), and 1 month and 3 months after the 2nd dose (7 weeks and 15 weeks after the first vaccine dose). Previous COVID-19 infection was ruled out by the clinical history and negative results for anti-SARS-CoV-2 N, S/RBD antibodies in samples collected immediately prior to the first vaccination. All participants provided written informed consent to use their samples for research purposes. The study is part of an ongoing single-center study approved by the Institutional Review Board at Seoul St. Mary’s Hospital (KC21TISI0114) to determine immunogenicity of different COVID-19 vaccines in HCWs. All participants were free of breakthrough infections until the time of last sample collection. Sampling times and intervals in participants are shown in **Figure 1**.

### 2.3 SARS-CoV-2 antibody assays

LABScreen™ COVID Plus Assay (One Lambda Inc., West Hills, CA, USA) is a multiplex semi-quantitative assay that detects antibodies against five SARS-CoV-2 targets: full S, S1, S2, RBD and N. For informational purposes, the panel also includes additional targets for seasonal coronaviruses, SARS, and MERS, namely HCoV-229E, HCoV-HKU1, HCoV-NL63, HCoV-OC43, MERS-CoV, and SARS-CoV. The assay consisted of 13 target protein-coated beads including negative and positive control beads. Antibodies on antigen-coated microparticles were detected using Luminex’s xMAP^®^ technology, according to the manufacturer’s instructions. Cut-off values provided by the manufacturer are 7,500 (anti-full S), 4,000 (anti-S1), 3,500 (anti-S2), 5,500 (anti-RBD), and 7,500 MFI (anti-N). Extra multiplex-bead assays containing additional microbeads coated with 23 different new variant spike antigens were performed in samples collected after COVID-19 infection (after 21 days from symptom onset) (n = 10) and 3 months after vaccination (AZ, n = 20 and BNT, n = 20).

Total anti-SARS-CoV-2 antibodies were also measured using the Roche Elecsys Anti-SARS-CoV-2 S chemiluminescent immunoassay, on a Roche Cobas e-801 (Roche Diagnostics, Basel, Switzerland). The assay cut-off is 0.8 U/mL with a linear quantification of detected results in the range of 0.4 – 250 U/mL by the manufacturer. If the measured levels exceeded 250 U/mL, samples were retested after additional dilution steps. The SARS-CoV-2 surrogate virus neutralization test (sVNT) (GenScript, Netherlands) was used to detect neutralizing antibodies targeting the RBD based on antibody-mediated blockage of the interaction between the ACE2 receptor and SARS-CoV-2 RBD. A cut-off of ≥30% inhibition was applied according to the manufacturer’s instructions. In the case of vaccinated individuals, to rule out previous infection, pre-vaccination samples were tested by STANDARD Q COVID-19 IgM/IgG plus (SD Biosensor Inc., Suwon-Si, Gyeonggi-do, Korea) detecting the anti-SARS-CoV-2 N, S/RBD antibodies.

### 2.4 Statistical analysis

Continuous data, given as median [interquartile range (IQR)], were compared by rank sign tests (Mann–Whitney U -test, Wilcoxon test). Categorical data are presented as counts and percentages, and data were compared with χ²-tests. Spearman rank correlation was used for comparison of quantitative values from different assay results, and correlations were defined as strong (0.7–1.0), moderate (0.5–0.7), and weak (0.3–0.5). Statistical analyses were performed using MedCalc 20.006 (MedCalc, Ostend, Belgium), and graphical representations of the data were performed using GraphPad Prism version 9.4.0 for Windows (GraphPad Software, San Diego, CA, USA). A p-value less than 0.05 was considered statistically significant.

## 3 Results

### 3.1 Seroconversion of SARS-CoV-2 antibody levels in COVID-19-infected patients and naïve vaccinated individuals

**Figure 2:**
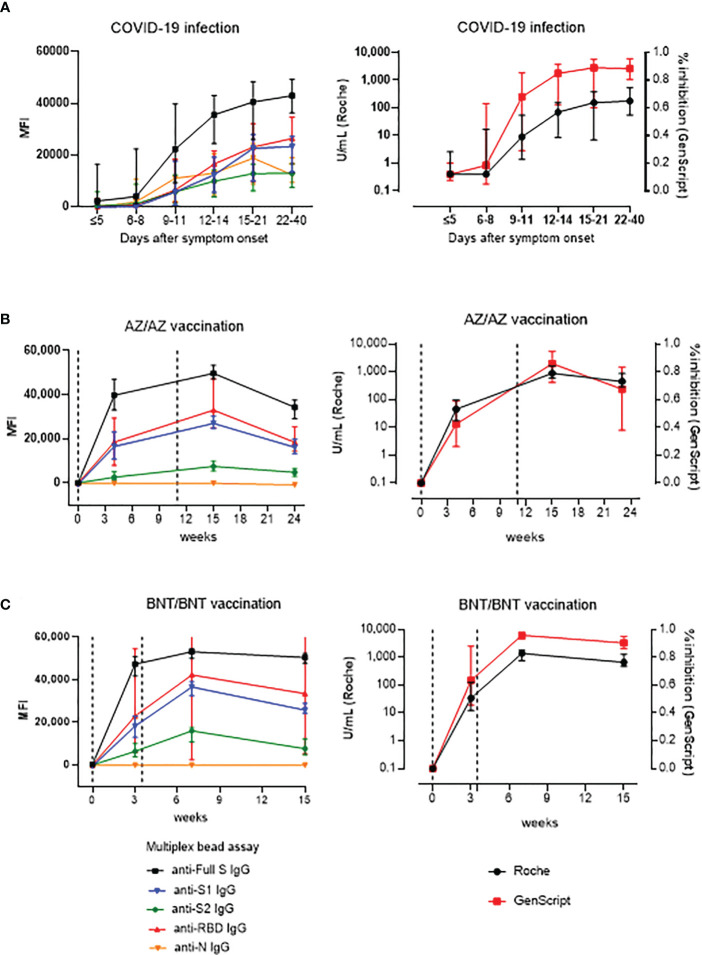
Kinetics of antibody response by multiplex (One-lambda), Roche and GenScript assay in naïve COVID-19 infection (n=32) **(A)**, AZ/AZ vaccination (n=20) **(B)** and BNT/BNT (n=20) vaccination **(C)**. X-axis shows the time point presented in days after symptom onset **(A)** and in weeks after the 1st-dose vaccination **(B, C)**. The 1st- and 2nd-dose vaccination time points are indicated by dotted lines. Data are presented as median and IQR.

Longitudinal serum samples from COVID-19 infected patients and vaccinated HCWs were tested for time course analysis of antibody responses (**Figure 2**). In COVID-19 patients, the multiplex-bead assay showed similar antibody levels against S1, S2, RBD, N antigens early in the infection (<14 days after symptom onset). However, after 2 weeks of infection, antibodies against full S, S1 and RBD showed higher median MFI values compared to those against S2 or N during, showing median levels of 42,542 MFI for anti-S, 23,113 for anti-S1, 25,796 for anti-RBD, 13,054 for anti-S2 and 13,123 for anti-N. Regarding the Roche assay, during 15-40 days after symptom onset, the antibody levels had a median (IQR) of 73.8 (3.4 – 354.3) and 183.5 (67.9 - 535.0) U/mL and positivity rates were of 95.2% and 96.3% for mild and severe disease patients, respectively. During this period, neutralizing antibody levels based on sVNT were 87.0% (45.5 – 93.3%) and 89.5% (84.0 – 96.0%) in mild and severe disease patients, respectively. When we compared antibody levels in 19 patients with severe course to 13 patients with mild disease course, anti-S, anti-S1 anti-S2, and anti-RBD antibody levels measured by multiplex-bead assay during 15-21 days were higher in patients with severe disease course compared to those with mild course (P < 0.05). Roche and GenScript assay results were not significantly different between mild and severe infections (P > 0.05) (**Supplemental Figure 1**). In vaccinated HCWs, SARS-CoV-2 antibody levels were monitored at time points from pre-vaccination to 3 months after boost vaccinations (until 24 weeks for AZ/AZ and 15 weeks for BNT/BNT) (**Figure 2B**). Both vaccine groups showed consistently high levels in the order of anti-full S, anti-RBD, anti-S1, and anti-S2, and no anti-N antibody was detected. In addition, the highest antibody levels were detected at 1 month after 2nd-dose vaccination.

### 3.2 Comparison of SARS-CoV-2 antibody levels between post-natural infection and post-vaccination

**Figure 3:**
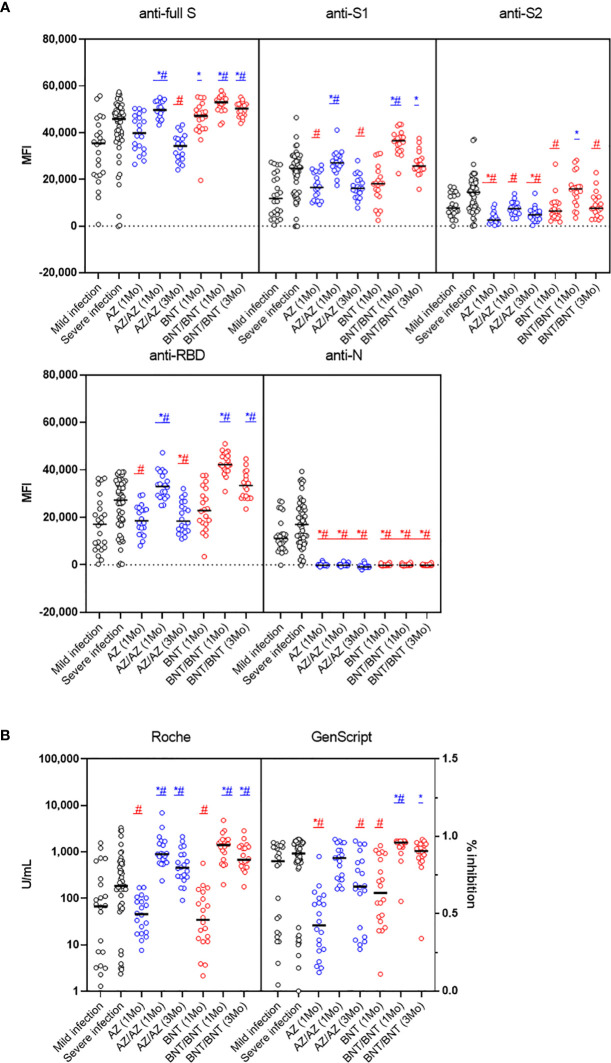
Comparison of early SARS-CoV-2 antibody levels by multiplex bead assay **(A)** and Roche and GenScript assays **(B)** in COVID-19 patients (>14 days from symptom onset) versus naïve vaccinated HCWs until 3 months after AZ or BNT boost vaccination. Blue markers (*, #) indicate higher and red markers indicate lower antibody levels in vaccination versus post-infection. *mild infection vs. vaccination (P < 0.05), #severe infection vs. vaccination (P < 0.05).

Antibody responses after mild or severe natural infection (>14 days from symptom onset) were compared with those after AZ or BNT vaccination (1MoA1D, 1MoA2D, 3MoA2D) (**Figure 3**). Among the five antibodies measured by multiplex-bead assay, anti-S2 and anti-N showed higher concentrations after natural infection than after vaccination (P < 0.05), except for anti-S2 levels 1 month after BNT vaccination. At one month after 2nd-dose vaccination with AZ or BNT, anti-full S, S1, RBD antibody levels by multiplex-bead assay and anti-S/RBD antibody levels by Roche assay were higher than after infection (P < 0.05). Three months after boost vaccination, the BNT group still showed higher antibody levels to full S, S1, RBD antigens compared to the infected patients (P<0.05). However, the AZ group 3 months after booster showed lower antibody levels than post-infection antibody levels by multiplex-bead assay and GenScript assay (P < 0.05). In contrast, Roche assay results were consistently higher in AZ or BNT vaccination until 3 months after the 2nd-dose vaccination than after infection.

### 3.3 Comparison of SARS-CoV-2 antibody levels measured by multiplex assay using new variant spike epitope-specific beads after natural infection and after 3-month boost vaccination

**Figure 4:**
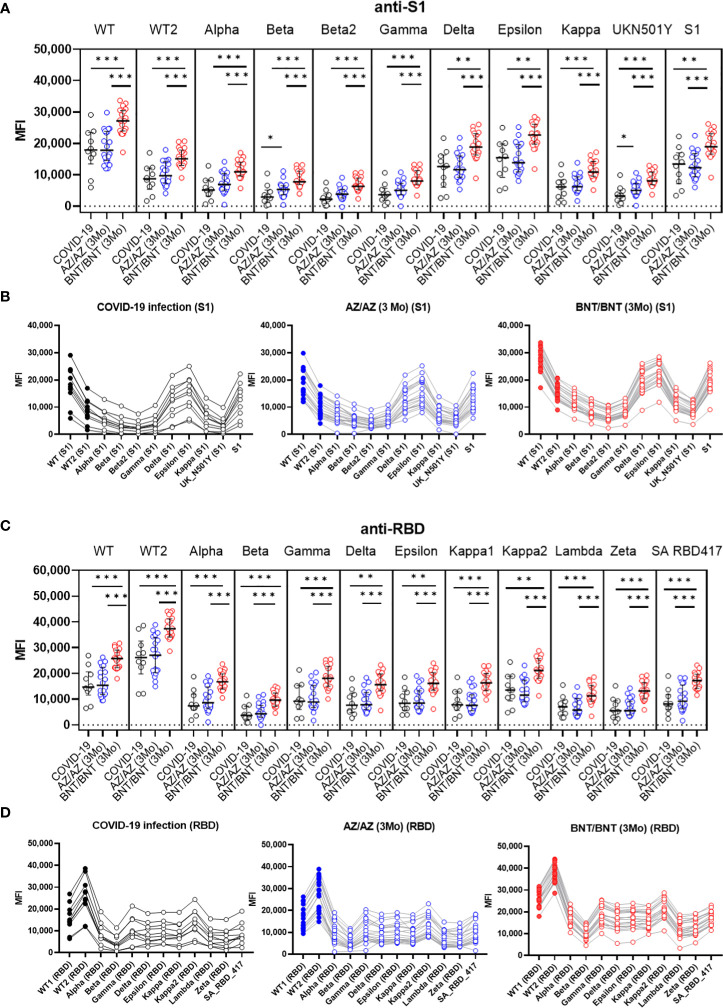
Comparison of anti-S1 **(A, B)** and anti-RBD **(C, D)** antibody levels against new variant spike antigens after COVID-19 infection (>21 days) (n=10) and 3 months after boost vaccination (AZ/AZ n=20, BNT/BNT n=20). Antibody levels were measured using microbeads coated with different new variant spike antigens (11 beads for anti-S1, 12 beads for anti-RBD) in a multiplex immunoassay. The same sera from 50 individuals were used in A-D. **(A)** BNT/BNT group (red) showed higher antibody levels than AZ/AZ group (blue) or COVID-19 patients (black) for all antibodies to different S1 antigens (P < 0.05). The AZ/AZ group showed higher levels of anti-S1 antibodies to beta S1 and UKN501Y antigens than those of COVID-19 patients (P < 0.05). **(B)** Comparison of antibody levels against different S1 antigens in three groups. **(C)** BNT/BNT group (red) showed higher antibody levels than AZ/AZ group (blue) or COVID-19 patients (black) for all antibodies to different RBD antigens (P < 0.05). **(D)** Comparison of antibody levels against different RBD antigens in three groups. *<0.5, **<0.01, **<0.001.

In limited numbers of samples from 10 COVID-19 patients (> 21 days from symptom onset) and 40 vaccinated HCWs (3MoA2D), anti-S and anti-RBD antibody levels against new variant spike epitopes were measured by multiplex-bead assay. Characteristics of new variant spike beads in LABscreen COVID plus kit are shown in **Supplemental Table 1**. Anti-S1 antibody levels against eleven S1 variant antigens and anti-RBD antibody levels against 12 RBD variant antigens were compared after COVID-19 and after 3-month boost vaccination (**Figure 4**). After 3 months boost vaccination of BNT showed higher anti-S1 and anti-RBD antibody levels against all 23 variant antigens than COVID-19 infection (P < 0.05). However, the AZ vaccine group (3MoA2D) did not differ from COVID-19 patients in antibody levels to most variant antigens. Antibody levels to S1 beta and S1 UK N501Y antigen were higher in the AZ vaccine group compared to COVID-19 patients (P = 0.043 and P = 0.031, respectively). When comparing AZ and BNT boost vaccination (3MoA2D), all antibodies to the new variant spike antigens showed higher levels in the BNT vaccines (P < 0.05). Anti-S1 and anti-RBD antibody levels for variant antigens including Alpha, Beta, Gamma and Deltas were decreased compared to wild-type antigens in all three groups (post-infection, 3 months after AZ/AZ vaccination, 3 months after BNT/BNT vaccination). In addition, there was a tendency to differ between anti-S1 and anti-RBD antibody levels within the same group, especially for Gamma, Delta and Kappa variants.

### 3.4 Comparison of SARS-CoV-2 antibody levels between AZ and BNT vaccination

**Figure 5:**
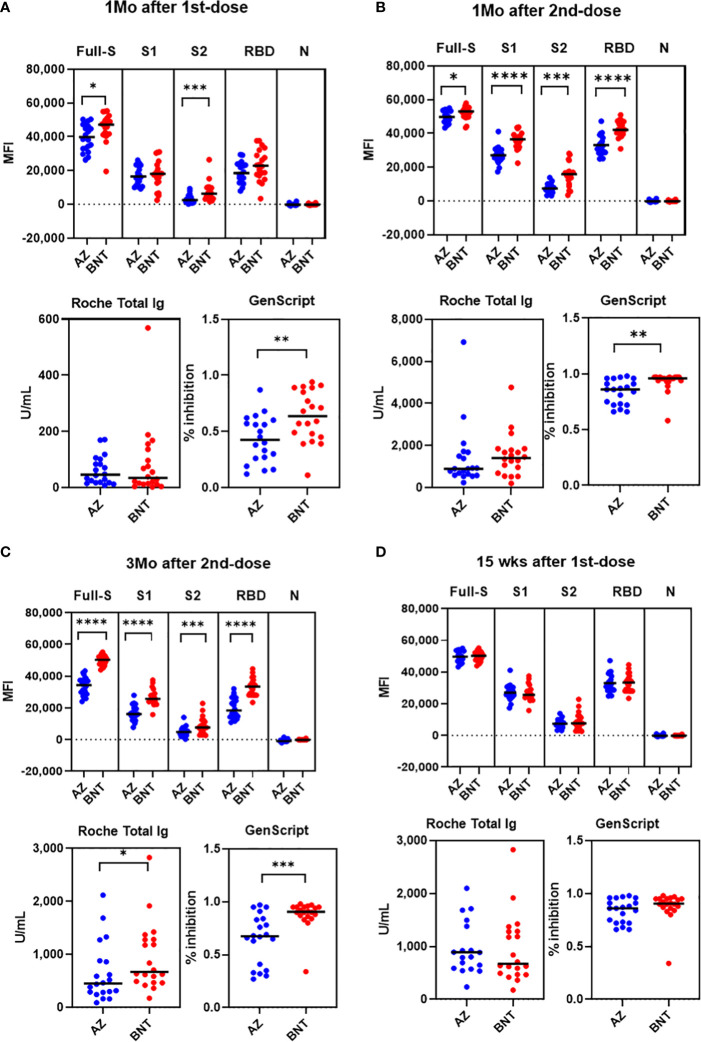
Comparison of SARS-CoV-2 antibody levels by Luminex bead assays, Roche and GenScript assays between AZ and BNT vaccination 1 month after 1st-dose **(A)**, 1 month after 2nd-dose **(B)**, 3 months after 2nd dose **(C)** and 15 weeks after 1st dose **(D)**. *P < 0.05, **P < 0.01, ***P < 0.001, ****P < 0.0001.

Next, we compared antibody levels at matched sampling times between AZ and BNT vaccination. Overall, BNT vaccination showed a trend for higher antibody levels in multiplex-bead assay and GenScript assay. After 1st-dose vaccination, anti-full S and anti-S2 levels were higher in BNT than in AZ vaccination (P < 0.05). However, anti-S1 and anti-RBD antibody levels were not different between AZ versus BNT vaccination ((anti-S1; 16,528 vs. 18,278 (AZ vs. BNT), anti-RBD; 18,506 vs. 22,865) (P>0.05) (**Figure 5A**). At this time, all vaccinated individuals showed anti-S RBD total Ig-positive (> 0.80 U/mL) in the Roche assay, and there was no difference between AZ and BNT vaccines (median of 45.7 U/mL and 34.3 U/mL, respectively) (P > 0.05). Regarding the neutralizing antibody by GenScript assay, 80.0% (16/20) of AZ and 95.0% (19/20) of BNT prime vaccinated HCWs had positive results (>30.0% inhibition) with higher neutralizing antibody levels in BNT versus AZ vaccination (median of 63.5% vs. 42.5%, P = 0.007) (**Figure 5A**).

After 1-month boost vaccination, anti-full S, S1, S2 and RBD antibody levels by multiplex assay and neutralizing antibody levels were higher in BNT compared to AZ vaccination (P < 0.05). However, anti-S RBD total antibody levels by Roche assays were not different between BNT and AZ vaccination [891.0 (618.0-1589.0) vs. 1404.5 (822.0-1813.0) U/mL] (P > 0.05). All individuals after boost vaccination had neutralizing antibody positive (>30% inhibition) with higher levels in the BNT/BNT compared to the AZ/AZ vaccination group [96.0% (93.5 - 97.0%) vs. 86.0% (72.5 - 93.5%), P = 0.003] (**Figure 5B**). All anti-S, RBD antibody levels measured by multiplex, Roche and GenScript assays at 3 months post-boost vaccination were higher in BNT than in AZ vaccination (P < 0.05) (**Figure 5C**). Because the prime-boost interval was different between AZ and BNT vaccination, we compared antibody response at 15 weeks post-prime vaccination. At 15 weeks after 1st-dose vaccination, all antibody levels tested in this study were not different between AZ and BNT vaccination (P > 0.05) (**Figure 5D**).

### 3.5 Intraindividual changes in SARS-CoV-2 antibody levels after AZ or BNT vaccination

Next, we assessed intraindividual changes in SARS-CoV-2 antibody levels after AZ or BNT vaccination (**Supplementary Figure 2**). Boost vaccination increased antibody levels by an average of 1.1- to 3.9-fold compared to prime vaccination. Roche assay and GenScript assay showed a 22.8- to 24.2-fold and 1.5- to 2.0-fold increase, respectively, during prime-boost vaccination when we analyzed intraindividual changes of antibody levels between 1 month and 3 months after 2nd-dose vaccination. Antibody levels measured by multiplex assay, Roche or GenScript assay were significantly decreased at 3 months after boost vaccination with a 0.5- to 1.1-fold change (P < 0.05). However, antibody levels 3 months after the 2nd dose were still higher compared to those after prime vaccination, with an exception of anti-full S antibody levels from AZ/AZ vaccination. Roche assay results were still 13.0- to 19.7-fold higher 3 months after boost vaccination than after prime vaccination.

**Figure 6:**
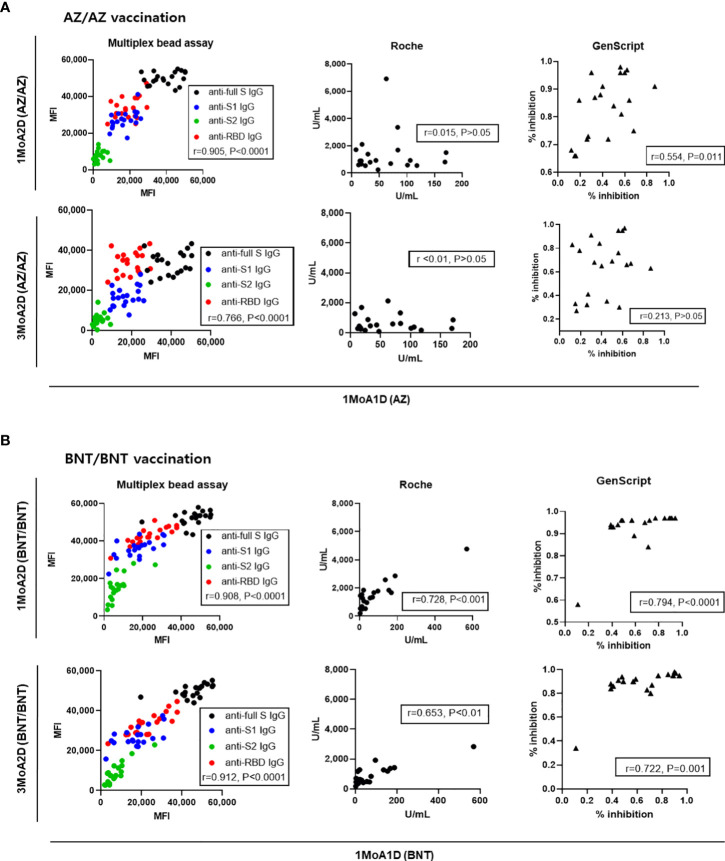
Correlation of antibody levels between post-prime and post-boost vaccination in 20 AZ/AZ **(A)** and 20 BNT/BNT **(B)** vaccinated health care workers.

To assess whether the antibody levels after prime vaccination (1MoA1D) were predictive of antibody levels after boost vaccination (1MoA2D or 3MoA2D), correlation analyses were performed. The antibody levels measured by multiplex-bead assay showed good correlation between after prime (1MoA1D) and after boost vaccination (1MoA2D, 3MoA2D) (r = 0.766 - 0.912, P < 0.0001) in both AZ and BNT vaccines (**Figure 6**). Regarding Roche and GenScript assay results between after prime and after boost vaccination, BNT vaccination showed strong correlation (r = 0.653 - 0.794, P < 0.01), however, AZ vaccination showed weak or no correlation (r = <0.010 - 0.554).

### 3.6 Correlation between binding antibody levels by multiplex-bead assay and Roche assay and neutralizing antibody levels by GenScript (sVNT)

**Figure 7:**
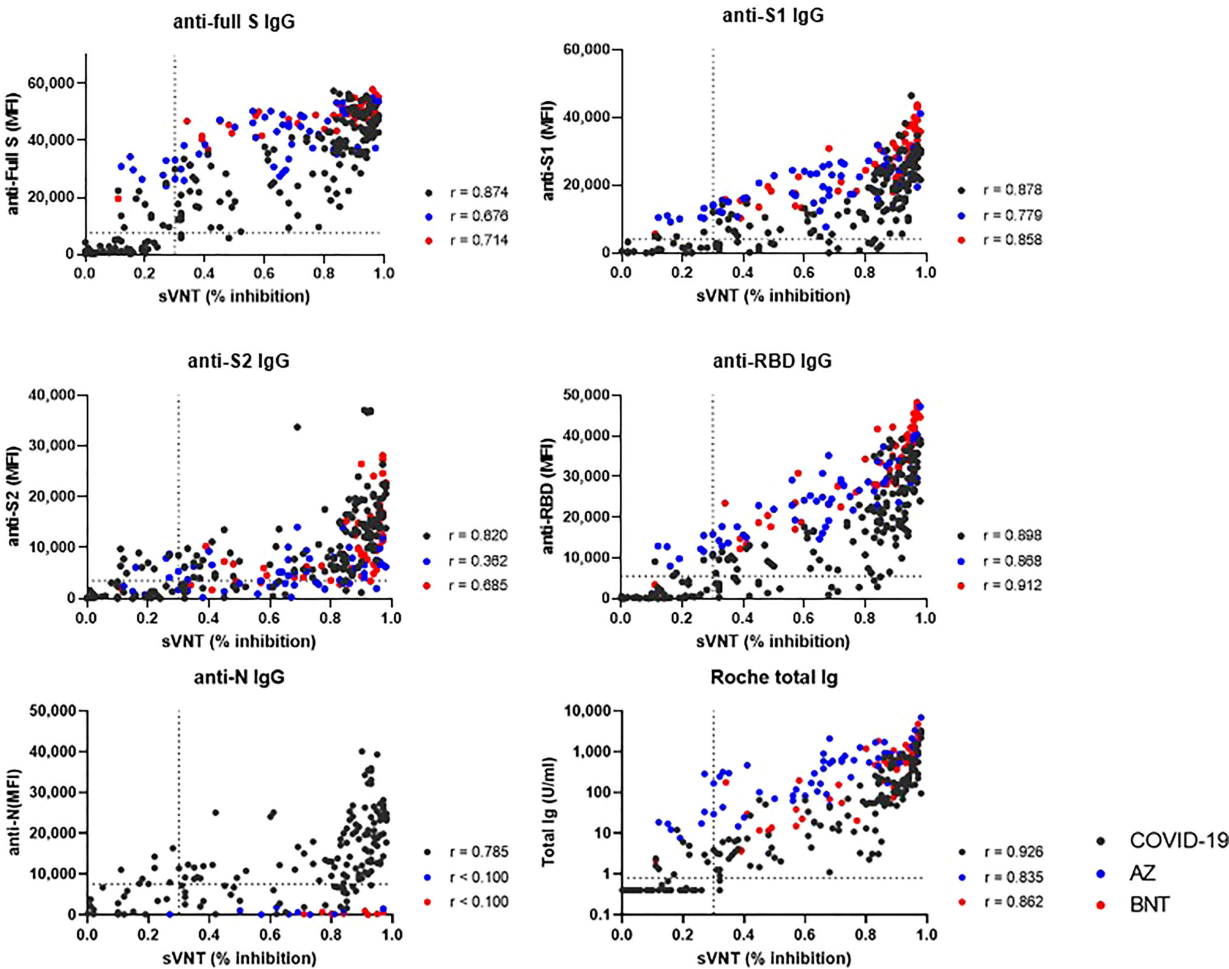
Correlation between binding antibody levels (anti-full S, S1, S2, RBD and N by luminex-bead assay and anti-S1 RBD total Ig by Roche assay) and neutralization antibody levels by GenScript assay. COVID-19 patients (black dots), AZ vaccine group (blue dots) and BNT vaccine group (red dots) are indicated. Spearman correlation coefficients (*r*) and cut-off levels (dotted lines) are displayed.

We compared binding antibody levels from multiplex-bead assay and Roche assay to neutralizing antibody levels of sVNT in COVID-19 patients, AZ and BNT groups (**Figure 7**). In COVID-19 patients, anti-full S, S1, S2, RBD and N levels and total Ig levels showed a strong correlation with the sVNT results (r = 0.785 – 0.926). In vaccinated HCWs, anti-S1, RBD IgG and total IgG levels strongly correlated with sVNT results (r= 0.779-0.912). However, anti-S2 levels showed weak to moderate correlation (r = 0.362 in AZ group and r = 0.685 in BNT group).

## 4 Discussion

The multiplex-bead assay offers the possibility to simultaneously assess antibody levels against a variety of target antigens, including S, S1, S2, RBD, and N. In the present study, sequentially collected samples from naturally infected patients and vaccinated HCWs were tested for SARS-CoV-2 antibodies using the multiplex-bead assay in conjunction with commercially available single antigen platform assays. Assessment of multiple biomarkers may lead to more accurate serological stratification of individuals, either infected or vaccinated.

A combination of anti-S1, S2, RBD, N and neutralizing antibody assays can be used to differentiate naturally infected patients from vaccinated patients, or to differentiate severe infections from mild infections (16, 20). When comparing five SARS-CoV-2 antigen-specific antibody levels in infected patients, multiplex-bead assay showed similar antibody levels against S1, S2, RBD, N antigens in the early infection period, but anti-full S, S1 and RBD antibody levels were higher than anti-S2 or N antibody levels from 2 weeks after infection. Regarding the infection severity, multiplex-bead assay showed a trend for higher levels in severe infections for all five antigens. And, after 2 weeks from symptom onset, S/RBD antibody levels were significantly higher in severe infection, confirming the previous reports measuring S/RBD antibody levels using single antigen platform assays (18, 19, 21). Roche and GenScript also showed a trend toward higher values in severely ill patients, despite no statistical difference. These findings support previous reports showing heterogeneity of antibody responses in quantitative antibody assays and the novelty of multiplex-bead assays for sequential testing against multiple epitopes (19, 22, 23).

In sequential samples up to 3 months after 2nd-dose vaccination, the highest antibody levels were detected 1 month after the 2nd dose for both the AZ vaccine and the BNT vaccine, as in the previous studies (24, 25). Vaccination developed continuously high levels of anti-full S, RBD S1, S2 in that order and no anti-N antibody was detected by multiplex-bead assay. In vaccinated individuals, a multiplex-bead assay measuring five antigen-specific antibodies simultaneously can be useful for assessing breakthrough infection, disease severity and time course of vaccine effectiveness.

When comparing antibody levels between post-infection and post-prime or boost vaccination, as expected, boost vaccination induced higher anti-S or RBD antibody levels by multiplex-bead assay and Roche assay (P < 0.05). These findings confirm that immune responses against S1 or RBD protein following boost vaccination become more intense versus COVID-19 infection (21). However, prime vaccination induced decreased or similar humoral immune response compared to natural infection. These results are consistent with previous reports showing higher antibody levels after COVID-19 infection than after prime vaccination until day 22 (21). Our study showed that anti-S2 and anti-N antibody levels were higher after natural infection than after vaccination, especially AZ. This finding supports previous reports that natural infection induced antibody response predominantly against nucleocapsid, full length and S2 domain of spike, and convalescent SARS-CoV-2 individuals had generated S2-reactive IgG and memory B cells (26, 27). Because N proteins are not present in currently used vaccines, anti-N antibodies are detected after natural exposure to SARS-CoV-2 and can be used to differentiate previously exposed individuals within a vaccinated population. When we compared antibody levels by different assay, all binding antibodies including anti-full S, S1, S2, RBD and N levels showed a strong correlation with sVNT results in COVID-19 patients. In vaccinated HCWs, multiplex-bead assays and Roche assays targeting RBD or S1 showed strong correlation with sVNT, whereas anti-S2 or N showed weak or no correlation with sVNT results. These results confirm previous reports that anti-S1 or RBD binding antibody assays can be used as an alternative to commercially available sVNT in vaccinated individuals (10, 13).

After 3 months of boost vaccination, the BNT group, but not the AZ group, still had higher levels of anti-S/RBD and neutralizing antibodies than the highest post-infection level (P<0.05). These findings confirm the previous reports showing a robust BNT162b2 vaccine-mediated-humoral immune responses and requirement of booster vaccination (4, 10). However, the Roche assay showed higher levels up to 3 months after booster vaccination in both AZ and BNT groups compared to infected patients (P < 0.05). These findings suggest that clinical laboratories need to know these assay-dependent differences in antibody levels to interpret vaccine or infection-induced antibody response. Our results confirm the importance of functional assays and that not all antibodies developed after vaccination or infection have neutralizing activity (13).

Previous studies have found that variants of concern are responsible for breakthrough infections in healthy individuals who received two-dose vaccination, particularly when antibody levels decrease over time (2, 4, 28). Mutations in the Beta, Gamma and Delta variant spike RBDs have been shown to induce partial resistance to neutralization by antibodies in convalescent and vaccinated individuals (2, 29, 30). Here, we found that SARS-CoV-2 infection or vaccination (AZ or BNT) also induced S- or RBD antibodies against new variant spike epitopes by testing multiplex-bead assay containing new variant spike epitope-specific beads. However, antibody responses against variant antigens are markedly decreased compared to those against wild-type S/RBD antigens. In addition, in sera collected 3 months post boost vaccination, the BNT group showed a high level of cross-neutralization, whereas the AZ group showed a significant decrease in antibody levels compared to BNT vaccination. These results are similar to previous reports showing decreased AZ-elicited antibody response against variants compared to BNT and support the rationale for booster immunization with BNT rather than AZ (4, 31). Furthermore, anti-S1 and anti-RBD antibody levels against variants showed different trends within the same group. This may be due to heterogeneity of antibody responses to RBD and S1 or variation in assay design including protein source and coating method. The mutant spike protein increased the infectivity of the pseudo-virus compared to the wild-type spike protein. Our findings by multiplex-bead assay support the importance of surveillance for breakthrough infections caused by new variants.

Next, we compared antibody responses between BNT and AZ vaccination. After 1st-dose vaccination, anti-full S, S2 and neutralizing antibody levels were higher in the BNT group than in the AZ group. This finding is consistent with a previous report that a single dose of AZ resulted in lower absolute antibody levels and slower responses compared with a single dose of BNT (24). However, in the present study, anti-S1, RBD IgG levels by multiplex assay and anti-S RBD total Ig levels by Roche assay were not different between the 1st doses of the AZ and BNT vaccines. These differences are probably due to differences in the target antigens in the assay and differences in the sensitivity of the assays used. After 2nd-dose vaccination, the AZ vaccine showed lower levels of anti-full S, S1, S2, RBD IgG and neutralizing antibodies than the BNT vaccine, which is in line with previous reports (32). However, when we compare antibody responses after 15 weeks vaccination, there was no difference in antibody levels including neutralizing antibody between AZ and BNT recipients. Therefore, although the AZ vaccine showed reduced antibody response relative to the BNT vaccine after 2nd-dose vaccination, time course analysis of antibody response revealed similar levels at 15 weeks after the 1st dose. This may be due to the different prime-boost interval between the AZ and BNT vaccines, and the fact that our study includes relatively younger HCWs compared to other cohorts (32). Why two-dose vaccination with the AZ vaccine yields lower antibody levels is not yet clear. A possible explanation could be the immune response to the adenovirus vector backbone and different vaccine schedule (10).

In the analysis for intraindividual changes of antibody levels, the rate of decrease in antibody levels was dependent on the assay method. The reduction of anti-RBD (multiplex assay) and neutralizing antibody (GenScript) from 1 month to 3 months after boost vaccination was more evident in the AZ vaccination than the BNT vaccination. When we correlated antibody response after prime vaccination to those after boost vaccination, the multiplex-bead assay showed good correlation between post-prime and post-boost response (r = 0.766-0.912) in both AZ and BNT vaccination. However, in the AZ vaccination group, post-prime responses by Roche and GenScript assay did not predict post-boost responses (r= <0.010 – 0.554). This finding has been reported by Peckmann et al. and they suggested the specific immune response to vector vaccines (11). Our data confirm this explanation, but these differences may not be found in multiplex-bead assay.

Regarding isotypes, immune system production of isotypes is time-dependent and is variously associated with virus neutralization (33). Previous studies have shown that IgM and/or IgA may be a major component of serum neutralization in the early convalescent period, and the anti-RBD and S1 IgG levels were significantly higher in post-vaccination cohort due to boosting immune exposures (34, 35). Unfortunately, we did not have the ability to test isotypes by multiplex bead assays. Further studies are needed to evaluate whether the various antibody responses, including isotypes by different assays, are associated with protection against clinical outcomes.

Limitations of the present study include the restricted cohort size due to the requirement of individuals who donate sequential samples, and the absence of samples from patients collected more than 40 days after symptom onset. Study limitations also include no data on underlying disease of participants and post-vaccination protection, and insufficient data to analyze antibody response over 3 months after 2nd dose AZ vaccination. In addition, culture-based neutralization antibodies using plaque reduction neutralization test, T-cell responses and binding antibodies to new variants including Omicron variant were not achieved in this study.

In summary, we presented comparative kinetics of SARS-CoV-2 antibodies against various epitopes in COVID-19 infection or vaccination. Our study suggests that one dose of AZ or BNT vaccination would be insufficient and shows lower anti-S, RBD IgG and neutralizing antibodies than peak levels in COVID-19-infected patients. All participants showed lower antibody levels against new variant antigens, which were dominant in the AZ vaccination group. The kinetics of SARS-CoV-2 antibody levels vary depending on the antigenic epitopes as well as assay kits, disease severity and vaccine type. Assessing seroconversion using multiplex-bead assays may contribute to monitoring the disease course, adjusting vaccination strategies, and accelerating vaccination efficacy.

## Data availability statement

The original contributions presented in the study are included in the article/**Supplementary Material**. Further inquiries can be directed to the corresponding author.

## Ethics statement

The studies involving human participants were reviewed and approved by Institutional Review Board at Seoul St. Mary’s Hospital (KC20SISI0879 & KC21TISI0114). The patients/participants provided their written informed consent to participate in this study.

## Author contributions

JL and D-GL wrote the manuscript, JJ, SS, S-YC, and RL supervised specimen selection and the collection of clinical information. JL and JR performed analyses. E-JO designed the study and wrote the manuscript. All authors contributed to the article and approved the submitted version.

## Supplementary material

Supplementary Figure 1 Seroconversion of SARS-CoV-2 antibodies by multiplex-bead assay (A) and Roche, GenScript assay (B) in patients with COVID-19 severe (solid line) and mild infection (dotted line). * P<0.05, ** P<0.01

Supplementary Figure 2 Intraindividual changes in SARS-CoV-2 antibody levels by multiplex-bead assay (A) and Roche or GenScript assay (B) after AZ and BNT vaccination. Fold change indicates the difference between antibody levels 1 month after 1st-dose (1MoA1D), 1 month after 2nd-dose (1MoA2D) and 3 months after 2nd-dose vaccination (3MoA2D).
