# Supplementary material for: Comprehensive assessment of SARS-CoV-2 antibodies against various antigenic epitopes after naive COVID-19 infection and vaccination (BNT162b2 or ChAdOx1 nCoV-19)
Source: Front Immunol. 2022 Dec 12;13:1038712. doi: 10.3389/fimmu.2022.1038712 (PMC9791030; doi:10.3389/fimmu.2022.1038712)
Supplement: Supplementary file 1 [file Table_1.docx]

Supplemental Table 1. Characteristics of new variant spike beads in LAbscreen COVID Plus kit

| Bead No. | S1/RBD | target epitope | Cat no. | VOC | Title in figure |
| --- | --- | --- | --- | --- | --- |
| 6 | S1 | S1 | 40591-V08H | WildType | WT |
| 16 | S1 | S1 | 40591-V08H | WildType | WT2 |
| 18 | S1 | Spike S1 (HV69-70 deletion, Y144 deletion, N501Y, A570D, D614G, P681) | 40591-V08H12 | Alpha | Alpha |
| 17 | S1 | Spike S1 (K417N, E484K N501Y, D614G) | 40591-V08H10 | Beta | Beta |
| 26 | S1 | Spike S1 (L18F, D80A, D215G, LAL242-244 deltion, R246I, K417, E484K, N501Y) | 40591-V08H15 | Beta2 | Beta2 |
| 25 | S1 | Spike S1 (L18F, T20N, P26S, D138Y, R190S, K417T, E484K, N501Y, D614G, H655Y) | 40591-V08H14 | Gamma | Gamma |
| 66 | S1 | SARS-Cov-2 Spike S1 (T19R, G142D, E156G, 157-158 deletion, L452R, T478K, D614G, P681) Protein (His Tag) | 40591-V08H23 | Delta S1 | Delta |
| 28 | S1 | Spike S1 (W152C, L452R, D614G) | 40591-V08H17 | Epsilon | Epsilon |
| 30 | S1 | Spike S1 (E154K, L452R, E484Q, D614G, P681R) | 40591-V08H19 | Kappa | Kappa |
| 31 | S1 | Spike S1 (HV69-70 dele, N501Y, D614G) | 40591-V08H7 | UK N501Y | UK N501Y |
| 71 | S1 | SARS-Cov-2 Spike S1 (G75V, T76I, R246N, 247 deletion, 253 deletion, L452Q, F490S, D614G) Protein His Taq | 40591-V08H31 |  | S1 |
| 33 | RBD | RBD | 40592-V08H | WildType | WT |
| 65 | RBD | RBD | 40592-V08H | WildType | WT2 |
| 43 | RBD | Spike RBD (N501Y) | 40592-V08H82 | Alpha (RBD) | Alpha |
| 83 | RBD | Spike RBD (K417N, E484K, N501Y) | 40592-V08H85 | Beta (RBD) | Beta |
| 88 | RBD | Spike RBD (K417T, E484K, N501Y) | 40592-V08H86 | Gamma (RBD) | Gamma |
| 92 | RBD | Spike RBD (L452R, T478K) | 40592-V08H90 | Delta (RBD) | Delta |
| 36 | RBD | Spike RBD (L452R) | 40592-V08H28 | Epsilon (RBD) | Epsilon |
| 39 | RBD | Spike RBD (E484Q) | 40592-V08H81 | Kappa (RBD) | Kappa1 |
| 89 | RBD | Spike RBD (L452R, E484Q) | 40592-V08H88 | Kappa (RBD) | Kappa2 |
| 75 | RBD | SARS-Cov-2 Spike RBD (L452Q, F490S) Protein His Taq | 45092-V08H113 | Lambda RBD | Lambda |
| 78 | RBD | Spike RBD (E484K) | 40592-V08H84 | Zeta (RBD) | Zeta |
| 96 | RBD | Spike RBD (K417N) | 40592-V08H59 | SA RBD 417 (RBD) | SA RBD 417 |
