# Supplementary figures and images for: Comprehensive assessment of SARS-CoV-2 antibodies against various antigenic epitopes after naive COVID-19 infection and vaccination (BNT162b2 or ChAdOx1 nCoV-19)
Source: Front Immunol. 2022 Dec 12;13:1038712. doi: 10.3389/fimmu.2022.1038712 (PMC9791030; doi:10.3389/fimmu.2022.1038712)

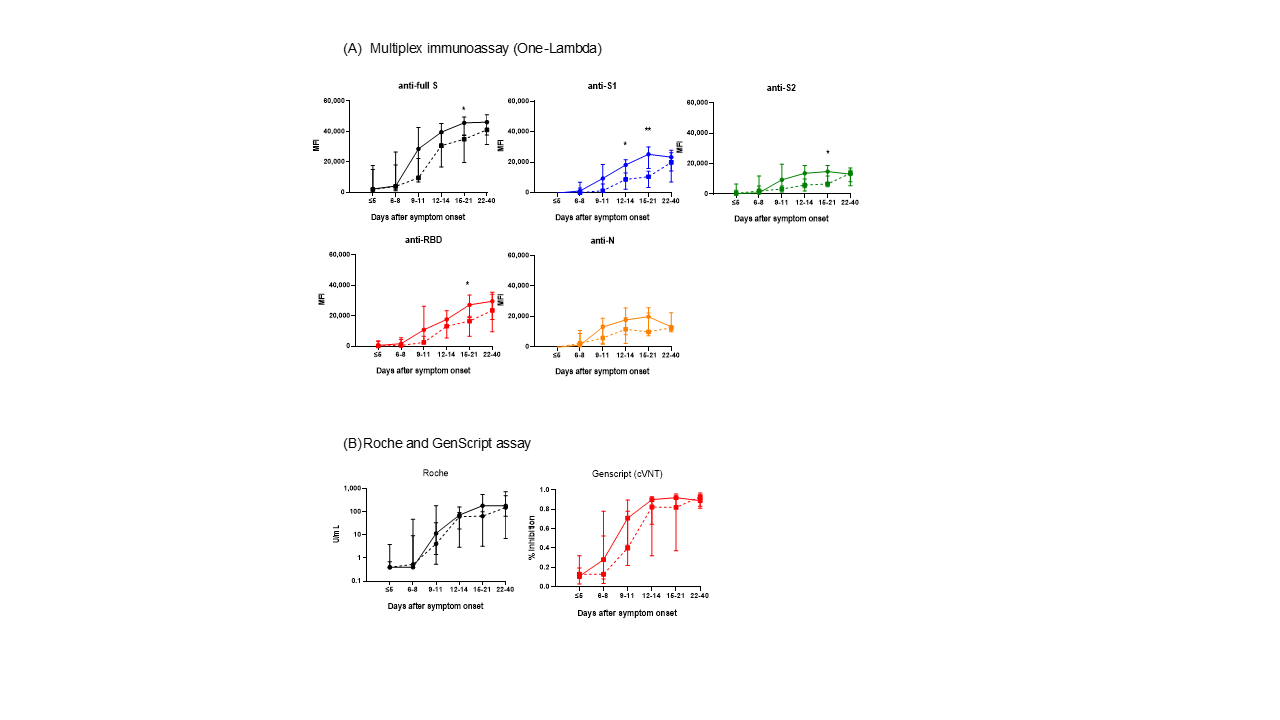

Supplement: Supplementary Figure 1 — Seroconversion of SARS-CoV-2 antibodies by multiplex-bead assay (A) and Roche, GenScript assay (B) in patients with COVID-19 severe (solid line) and mild infection (dotted line). * P<0.05, ** P<0.01 [file Image_1.tif]

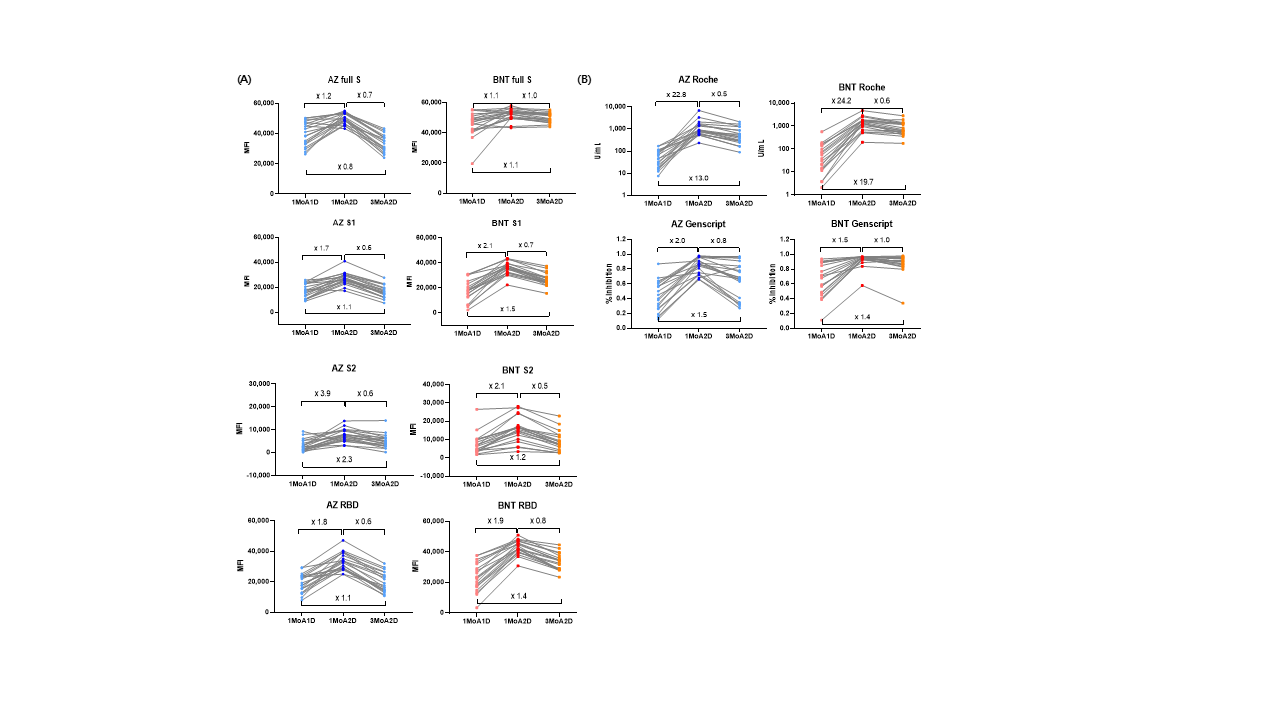

Supplement: Supplementary Figure 2 — Intraindividual changes in SARS-CoV-2 antibody levels by multiplex-bead assay (A) and Roche or GenScript assay (B) after AZ and BNT vaccination. Fold change indicates the difference between antibody levels 1 month after 1st-dose (1MoA1D), 1 month after 2nd-dose (1MoA2D) and 3 months after 2nd-dose vaccination (3MoA2D). [file Image_2.tif]
